# Structural Prioritization of FatB Thioesterase Candidates Potentially Related to Lauric Acid-Rich Seed Oil in *Litsea cubeba*

**DOI:** 10.3390/biom16060813

**Published:** 2026-05-30

**Authors:** Wenyan Yuan, Changzhu Li, Jingzhen Chen, Peiwang Li, Xiao Zhou, Wei Wu, Lijuan Jiang, Wenbin Zeng, Feng Wen, Yunzhu Chen, Yan Yang

**Affiliations:** 1 State Key Laboratory of Woody Oil Resources Utilization, Central South University of Forestry and Technology, 498 South Shaoshan Road, Changsha 410004, China; 20231100133@csuft.edu.cn (W.Y.); slky@hnlky.cn (C.L.); w2052065326@163.com (W.W.); znljiang2542@163.com (L.J.); csuft85658537@163.com (W.Z.); wen19992022@163.com (F.W.); 2 Hunan Academy of Forestry, Changsha 410004, China; chenjingzhen602@hnlky.cn (J.C.); lindan523@163.com (P.L.); zx924647234@163.com (X.Z.); 3 Institute of Urban Agriculture, Chinese Academy of Agricultural Sciences, Chengdu 610213, China; 4 Yuelushan Laboratory, Changsha 410000, China

**Keywords:** *Litsea cubeba*, lauric acid, FatB thioesterase, molecular dynamics, catalytic tunnel

## Abstract

Lauric acid is a characteristic component of *Litsea cubeba* seed oil, but FatB thioesterase candidates with predicted structural compatibility for C12 acyl-substrate accommodation remain insufficiently defined. In this study, seed oil content and fatty acid composition were examined during *L. cubeba* seed development. The fatty acid profile shifted from a C18:2-rich pattern at the early stage to a C12:0-dominated composition at later stages, providing the biochemical context for FatB candidate prioritization. Three FatB-like candidates were retrieved from a de novo seed transcriptome assembly and named LcFatB1, LcFatB2, and LcFatB3. Phylogenetic analysis, conserved motif comparison, sequence alignment, and homology modeling showed that LcFatB1 and LcFatB2 retained more complete FatB-like sequence and structural features than LcFatB3. S-dodecanoyl-4′-phosphopantetheine was used as a C12 acyl-4′-phosphopantetheine surrogate for molecular docking. Docking analysis indicated that LcFatB1 and LcFatB2 formed more interpretable C12-bound poses than LcFatB3. Subsequent 150 ns molecular dynamics simulations, free energy landscape analysis, residue–ligand interaction profiling, and catalytic tunnel analysis further distinguished the two main candidates. Compared with LcFatB2, LcFatB1 maintained a lower-displacement C12-bound state, a more compact contact environment involving Tyr116, Ser125, and Asn278, and a main tunnel with higher throughput and shorter length in the representative global-minimum conformation. LcFatB2 also retained the C12 surrogate but stabilized it in a distinct rearranged binding environment. These results support LcFatB1 as the strongest structurally prioritized FatB candidate among the three transcriptome-derived proteins, while LcFatB2 remains a plausible FatB-like candidate with a distinct C12-bound state. This prioritization provides computational structural clues for future biochemical testing but should not be interpreted as direct functional confirmation of FatB activity in vivo.

## 1. Introduction

*Litsea cubeba* is a Lauraceae woody oil plant whose seeds accumulate a considerable amount of medium-chain saturated fatty acids [1,2]. Among these fatty acids, lauric acid is particularly abundant and differs from the C16- and C18-dominated profiles commonly observed in many seed oils. This lauric acid-rich composition has also supported the consideration of *L. cubeba* kernel oil as a potential lauric oil resource for green extraction, refining, and integrated valorization [3,4]. This composition suggests that fatty acid chain termination in *L. cubeba* seeds is biased toward medium-chain acyl intermediates. However, the enzyme candidates potentially associated with this medium-chain fatty acid output have not been clearly defined.

In plastidial fatty acid synthesis, the growing acyl chain remains attached to acyl carrier protein until it is released by acyl-ACP thioesterases [5]. This hydrolytic step is critical because it terminates chain elongation and contributes directly to the fatty acid profile of seed oil [6]. Recent functional studies of soybean FATA genes also support the essential role of acyl-ACP thioesterases in fatty acid accumulation and plant growth [7]. Other oil-producing plant studies have further linked FAT gene regulation with seed or fruit oil fatty acid composition [8]. Plant acyl-ACP thioesterases are generally divided into FatA and FatB classes. FatA enzymes preferentially act on unsaturated C18 acyl-ACPs, whereas FatB enzymes are more closely associated with saturated acyl-ACP hydrolysis [9,10]. In species that accumulate unusual saturated fatty acids, FatB members often serve as the primary candidates for explaining chain-length specificity. Recent analyses of plant FAT genes have also linked this gene family to seed oil content, fatty acid composition, expression divergence, and regulatory networks in oil-producing or cereal crops [11,12]. Functional analysis of soybean FATB genes further showed that FATB overexpression can alter seed oil content and fatty acid profiles in transgenic *Arabidopsis*, supporting the relevance of FatB-type enzymes to seed lipid composition [13]. Accordingly, FatB thioesterases represent a relevant enzyme family for prioritizing candidates potentially linked to lauric acid accumulation in *L. cubeba*.

Sequence annotation alone is insufficient to infer whether a FatB-like candidate is associated with C12 acyl-substrate accommodation or C12:0 accumulation [14]. FatB-like proteins may share conserved motifs and similar catalytic folds, but small differences in pocket shape, residue placement, and substrate access routes can alter acyl-chain accommodation [15]. For a C12 acyl substrate, productive binding requires not only placement of the thioester region near the catalytic site, but also appropriate packing of the hydrocarbon chain within the binding cavity [16,17]. The path connecting the pocket to the protein surface may also influence substrate entry or product release [18]. Therefore, candidate prioritization requires structural information beyond phylogenetic clustering and motif conservation. However, for *L. cubeba*, FatB-like candidates have not been structurally compared in relation to C12 acyl-substrate accommodation. It remains unclear which candidate has a binding pocket, a residue-contact pattern, and a substrate-access route that are more compatible with C12 substrate placement.

Ligand definition is another important issue in structure-based analysis of FatB candidates. The native substrate of FatB is acyl-ACP rather than free lauric acid. The ACP component is not only a passive carrier but also contributes to acyl-chain handling and enzyme recognition in fatty acid biosynthesis [19]. Docking free lauric acid removes the phosphopantetheine arm and the thioester linkage, both of which are part of the natural acyl-ACP substrate. Recent work has also shown that the acyl carrier protein portion can influence the chain-length preference of acyl-ACP thioesterase, further supporting the need to distinguish acyl-ACP-like substrates from free fatty acids [20]. Conversely, full lauroyl-ACP introduces a large and flexible carrier protein component that is not suitable for routine small-molecule docking. Therefore, an appropriate substrate representation is needed to examine C12 acyl-chain accommodation in the FatB binding pocket.

In this study, we aimed to characterize seed oil accumulation and fatty acid composition during *L. cubeba* seed development and to structurally prioritize FatB-like candidates potentially related to C12:0 accumulation. To achieve this aim, seed oil content and fatty acid composition were first determined at representative developmental stages. FatB-like candidates were then retrieved from a de novo seed transcriptome assembly and evaluated using phylogenetic analysis, conserved motif comparison, sequence alignment, and homology modeling. To further examine their predicted compatibility with a C12 acyl substrate, molecular docking, molecular dynamics simulation, free energy landscape analysis, residue–ligand interaction profiling, and catalytic tunnel analysis were performed.

## 2. Materials and Methods

### 2.1. Plant Materials and Seed Sampling

Fruits of *Litsea cubeba* were collected from the *L. cubeba* germplasm repository at the Shigongpo Experimental Forest Station of the Hunan Academy of Forestry, China. The sampled material belonged to the elite cultivar *L. cubeba* No. 2. Fruit collection was carried out during seed development, beginning at 50 days after flowering (DAF) and continuing until physiological maturity at 160 DAF. The developmental stages used for oil content and fatty acid profiling in this study were 50, 80, 110, 140, and 160 DAF.

After collection, the fruits were immediately processed in the laboratory. The pericarp was manually removed, and the seeds were separated for subsequent analyses. For oil content determination, seed samples were dried in a forced-air oven (Thomas Scientific, Swedesboro, NJ, USA) at 70 °C until a constant weight was reached. For fatty acid composition analysis, another portion of the seeds was rapidly frozen in liquid nitrogen and stored at −80 °C before use. These sampling and storage procedures were used to minimize post-harvest changes in seed lipid composition and to maintain the biochemical state of the developing seeds.

### 2.2. Determination of Seed Oil Content

Seed oil content was determined using Soxhlet extraction according to the Chinese National Standard GB 5009.6-2016 [21], with three independent extractions performed for each developmental stage. Dried seed samples were ground into a fine powder, and 2–5 g of each sample was accurately weighed as the initial dry mass. The powdered sample was transferred to a fat analyzer (CEM Corporation, Matthews, NC, USA) and extracted with petroleum ether (boiling range, 30–60 °C) at 65 °C for 6 h. After extraction, the remaining residue was dried at 105 °C for 2 h, cooled in a vacuum desiccator for 30 min, and weighed after reaching room temperature. Seed oil content was calculated from the mass loss after solvent extraction and expressed as a percentage of seed dry weight.

The oil content was calculated using the following equation:

$$Oil\;content\;\left(\%\right)=\frac{M_{0}-M_{1}}{M_{0}}\times 100\%$$

where $M_{0}$ represents the initial dry mass of the seed powder before extraction, and $M_{1}$ represents the dry mass of the residue after extraction.

### 2.3. Seed Oil Extraction and Preparation of Fatty Acid Methyl Esters

Seed oil used for fatty acid composition analysis was extracted from the corresponding *L. cubeba* seed samples before fatty acid methyl ester preparation. Briefly, seed samples from each developmental stage were dried and ground into a fine powder. The powdered samples were extracted with petroleum ether (boiling range, 30–60 °C) under the same solvent-extraction conditions used for seed oil content determination. After extraction, the solvent was removed, and the recovered seed oil was stored at −20 °C under sealed conditions until derivatization.

Fatty acid methyl esters (FAMEs) were prepared from the extracted *L. cubeba* seed oil before gas chromatography analysis. Briefly, approximately 0.06 g of seed oil was weighed into a 10 mL centrifuge tube and dissolved in 4 mL of isooctane by vortexing for 2 min. Then, 200 μL of 2 mol/L potassium hydroxide in methanol was added, and the mixture was vigorously vortexed for 60 s to initiate alkaline transesterification. The reaction was allowed to proceed briefly at room temperature, and the total contact time after addition of potassium hydroxide in methanol was controlled within 3 min to avoid prolonged alkaline treatment of the methyl esters. Subsequently, 1 g of anhydrous sodium hydrogen sulfate was added to terminate and neutralize the reaction, followed by vortexing for 30 s. After standing for 10 min, the upper organic phase containing FAMEs was collected and passed through a 0.22 μm organic membrane filter for subsequent GC analysis.

### 2.4. Gas Chromatography Analysis of Fatty Acid Composition

The prepared FAME samples were analyzed using a Nexis GC-2030 gas chromatograph equipped with a flame ionization detector (Shimadzu, Kyoto, Japan). Separation was performed on a fused-silica capillary column (100 m × 0.25 mm, 0.25 μm film thickness; Supelco/Sigma-Aldrich, Bellefonte, PA, USA). Nitrogen was used as the carrier gas at a constant flow rate of 1.1 mL/min, and the split ratio was set to 1:100. The injection volume was 1.0 μL, and the injector temperature was maintained at 270 °C.

The oven temperature program was set as follows: the initial temperature was held at 100 °C for 13 min, increased to 180 °C at 10 °C/min and held for 6 min, then increased to 200 °C at 1 °C/min and held for 20 min, and finally raised to 230 °C at 4 °C/min and held for 10.5 min. Fatty acid methyl esters were identified by comparing their retention times with those of the Supelco 37 Component FAME Mix standard (Sigma-Aldrich/Supelco, St. Louis, MO, USA). The relative abundance of each major fatty acid was calculated using peak-area normalization and expressed as a percentage of the total peak area of the major fatty acids shown in this study. The isooctane solvent peak was excluded from the quantification of fatty acid composition.

### 2.5. Transcriptome Data Source and Retrieval of FatB Candidates

The raw RNA-seq reads of *Litsea cubeba* seeds were obtained from the Genome Sequence Archive of the China National Center for Bioinformation under the accession number CRA021915. These reads were originally generated from developing *L. cubeba* seeds and were used here as the sequence source for FatB candidate retrieval. Because the deposited data correspond to raw transcriptome reads rather than transcript-level accessions, FatB candidates in this study were retrieved from the de novo seed transcriptome assembly generated from these reads.

Transcriptome-derived gene catalogs have been widely used as sequence resources for candidate gene retrieval in non-model plants. Putative FatB transcripts were first searched from the seed transcriptome assembly using reported plant FatB proteins as queries. Candidate retention was based on four criteria: FatB-like annotation, the presence of conserved acyl-ACP thioesterase regions, ORF and protein-length interpretability, and suitability for downstream structural modeling and docking analysis. Candidate sequences were retained when their annotations and conserved regions were consistent with acyl-ACP thioesterase B-like proteins. Sequences with extremely short or poorly interpretable coding regions were excluded from the main candidate set. After this screening, three FatB-like transcripts were retained for comparative analysis and named LcFatB1, LcFatB2, and LcFatB3. Among them, LcFatB1 and LcFatB2 showed more suitable sequence completeness and protein length for subsequent structure-based comparison, whereas LcFatB3 was retained as a shorter supplementary FatB-like candidate. Their original transcriptome assembly IDs were TRINITY_DN9967_c0_g1_i3.p1, TRINITY_DN5292_c3_g1_i1.p1, and TRINITY_DN17457_c0_g1_i4.p1, respectively. These assigned names were used throughout the manuscript to simplify sequence description and figure labeling.

### 2.6. Phylogenetic Analysis

To determine the evolutionary placement of the three LcFatB candidates, reference plant FatB protein sequences were retrieved from public protein databases and used together with LcFatB1, LcFatB2, and LcFatB3 for phylogenetic analysis. The reference set included FatB or FatB-like acyl-ACP thioesterases from representative plant species, with accession numbers Q39513.1, AEM72522.1, AAC49269.1, AGG79285.1, NP_172327.1, AEM72521.1, AEM72520.1, Q39473.1, and Q41635.1. Protein sequences were aligned using the ClustalW algorithm implemented in MEGA 11. Poorly aligned terminal gaps were checked manually before tree construction. A phylogenetic tree was constructed using the maximum likelihood method in MEGA 11, and node support was evaluated by bootstrap analysis with 1000 replicates [22]. Bootstrap percentage values were used to assess the reliability of internal branches. The resulting tree topology was used to examine the placement of LcFatB candidates relative to reported plant FatB proteins.

### 2.7. Conserved Motif and Sequence Logo Analysis

Conserved motifs were identified using the MEME Suite (https://meme-suite.org/meme/tools/meme, accessed on 17 March 2026) [23]. The same protein sequences used for phylogenetic analysis were subjected to motif detection to ensure consistency between sequence clustering and motif comparison. The maximum number of motifs was set to eight. Motif discovery was performed using the amino acid sequences of all included FatB or FatB-like proteins, and motif positions were mapped according to their coordinates in each protein sequence.

Sequence logos were generated from the identified motifs to display residue conservation at each position. The height of each amino acid in the logo represents its relative conservation. Motif distribution and sequence logo results were then used for comparative analysis of conserved sequence features among the LcFatB candidates and reference FatB proteins.

### 2.8. Multiple Sequence Alignment

Multiple sequence alignment of LcFatB1, LcFatB2, and LcFatB3 was performed using ESPript 3.2 (https://espript.ibcp.fr/ESPript/ESPript/, accessed on 17 March 2026). A conserved region shared by the three candidates was selected for alignment visualization, with conserved and similar residues displayed according to the default ESPript residue-shading scheme. Pairwise alignment between LcFatB1 and LcFatB2 was also conducted using the same platform.

### 2.9. Homology Modeling and Model Validation

Homology models of LcFatB1, LcFatB2, and LcFatB3 were generated using the SWISS-MODEL server (https://swissmodel.expasy.org/). Template selection was based on sequence identity, sequence coverage, GMQE score, and predicted oligomeric state. LcFatB1 was modeled using the crystal structure of dodecanoyl-ACP thioesterase from *Umbellularia californica* as the template (5X04.1.A), with a sequence identity of 91.04% and a GMQE score of 0.69. The selected template was a homodimer, and the corresponding dimeric assembly was retained for LcFatB1 modeling. LcFatB2 was modeled using the AlphaFold DB model of FATB1 from *Gossypium hirsutum* (Q9SQI3.1.A), with a sequence identity of 71.32% and a GMQE score of 0.73. LcFatB3 was modeled using the AlphaFold DB model of an acyl-ACP thioesterase from *Papaver somniferum* (A0A4Y7JJN6.1.A), with a sequence identity of 73.27% and a GMQE score of 0.87.

The stereochemical quality of the modeled structures was checked using Ramachandran plot analysis through the SAVES v6.1 server (https://saves.mbi.ucla.edu/). Backbone dihedral-angle distributions were inspected to evaluate the overall reasonability of the predicted models before subsequent structural comparison and docking analysis.

### 2.10. Molecular Docking

S-dodecanoyl-4′-phosphopantetheine was used as the C12 acyl-4′-phosphopantetheine surrogate for docking analysis. This ligand was selected to approximate key chemical features of the native acyl-ACP substrate while keeping the docking system tractable. It retains the dodecanoyl chain, thioester linkage, and phosphopantetheine group, and is therefore more representative of a C12 acyl-ACP-derived substrate than free lauric acid. Full lauroyl-ACP was not used because the carrier protein introduces a large and flexible protein–protein recognition component that is not suitable for routine small-molecule docking.

The modeled structures of LcFatB1, LcFatB2, and LcFatB3 were used as receptors. Receptor and ligand files were prepared using AutoDockTools v1.5.7, including hydrogen addition, Gasteiger charge assignment, and conversion to PDBQT format. Docking calculations were performed using AutoDock Vina v1.1.2 [24].

For each LcFatB model, the docking search space was centered on the predicted substrate-binding cavity and sized to cover the catalytic pocket and adjacent acyl-chain accommodation region. A cubic grid box of 50 × 50 × 50 Å was used for all docking calculations. The center of the search space was defined according to the geometric center of the predicted pocket region surrounding the conserved catalytic cavity in each modeled structure. Docking was performed with an exhaustiveness value of 24, and 10 binding modes were generated for each receptor–ligand pair within an energy range of 3 kcal/mol. The receptor was treated as rigid, whereas ligand rotatable bonds were kept flexible during docking.

Generated poses were first ranked according to the Vina score and then visually inspected for pocket-oriented placement. The representative pose was selected based on three criteria: a relatively favorable Vina score, location of the ligand within the predicted substrate-binding cavity, and an orientation in which the thioester/phosphopantetheine region was positioned toward the polar pocket region while the dodecanoyl chain extended into the acyl-chain accommodation region. Docked complexes were inspected using VMD, and two-dimensional protein–ligand interaction diagrams were generated using LigPlot+ v2.2.9 [25].

### 2.11. Molecular Dynamics Simulation and Trajectory Analysis

Molecular dynamics simulations were performed for the LcFatB1–S-dodecanoyl-4′-phosphopantetheine and LcFatB2–S-dodecanoyl-4′-phosphopantetheine complexes using GROMACS 2020.6 [26]. The CHARMM36 force field was used for the proteins, and ligand topology files were generated using the CGenFF server (https://cgenff.com/). Each complex was placed in a periodic solvent box and solvated with TIP3P water molecules. Counterions were added to neutralize the system. Energy minimization was conducted using the steepest-descent algorithm until the maximum force was below 1000 kJ mol^−1^ nm^−1^. The minimized systems were then equilibrated for 100 ps under NVT conditions and for 100 ps under NPT conditions. During equilibration and production simulations, temperature was maintained at 300 K using the velocity-rescaling thermostat, and pressure was maintained at 1 bar using the Parrinello–Rahman barostat. Long-range electrostatic interactions were treated using the particle-mesh Ewald method, and covalent bonds involving hydrogen atoms were constrained using the LINCS algorithm. Short-range electrostatic and van der Waals interactions were calculated with a cutoff of 1.0 nm. One 150 ns production simulation was performed for each complex with a 2 fs time step. Independent replicate simulations were not performed in the present study; therefore, the MD results were interpreted as comparative structure-based evidence rather than statistical confirmation of dynamic behavior.

Trajectory processing was performed after removal of periodic boundary effects, and each trajectory was fitted to the protein backbone. Backbone root-mean-square deviation, radius of gyration, solvent-accessible surface area, and protein–ligand hydrogen bond number were calculated to monitor the global behavior of the two complexes. Residue-level root-mean-square fluctuation (RMSF) was calculated for Cα atoms to evaluate local flexibility along the protein sequence, with particular attention to regions surrounding the predicted ligand-binding pocket. Representative structures were extracted at 0, 50, 100, and 150 ns for conformational inspection. The 0 ns and 150 ns structures were aligned by the protein backbone and rendered as superimposed views to compare the initial and final conformational states. Molecular structures and trajectory snapshots were visualized using VMD 1.9.3.

Free energy landscapes were constructed using protein RMSD and protein–ligand distance as reaction coordinates. The conformational probability distribution was converted into Gibbs free energy according to ΔG = −RT ln(*P*/*P*_max), where *P* represents the probability of a given conformational state and Pmax is the maximum probability observed in the trajectory. The global minimum basin was identified from each free energy landscape, and the corresponding representative structure was extracted for subsequent structural comparison.

Key residue interactions were analyzed using the representative structures extracted from the global minimum basins. Residues in contact with S-dodecanoyl-4′-phosphopantetheine in these structures were selected for interaction profiling. Hydrogen bond occupancy was calculated for the selected residues by tracing their hydrogen-bond formation with the ligand across the corresponding MD trajectory. The minimum heavy-atom distance between each selected residue and S-dodecanoyl-4′-phosphopantetheine was also measured to evaluate the persistence of residue–ligand contacts associated with the global-minimum binding mode.

Catalytic tunnel analysis was performed using CAVER v2.0 (https://loschmidt.chemi.muni.cz/caverweb/, accessed on 17 March 2026) based on the representative structures extracted from the global minimum basins of the LcFatB1 and LcFatB2 complexes. The tunnel starting point was defined near the ligand-binding cavity, using the bound S-dodecanoyl-4′-phosphopantetheine and adjacent pocket residues as references. Tunnels were clustered according to spatial similarity, and the main tunnel was selected based on throughput and its connectivity with the ligand-binding cavity. Tunnel throughput, bottleneck radius, length, and curvature were calculated to compare the substrate-access routes associated with the global-minimum conformations of LcFatB1 and LcFatB2. This analysis was designed as a representative-structure-based comparison and was not intended to describe trajectory-wide tunnel dynamics or cluster-dependent tunnel variation.

## 3. Results

### 3.1. Oil Accumulation and Fatty Acid Composition During Seed Development

**Figure 1:**
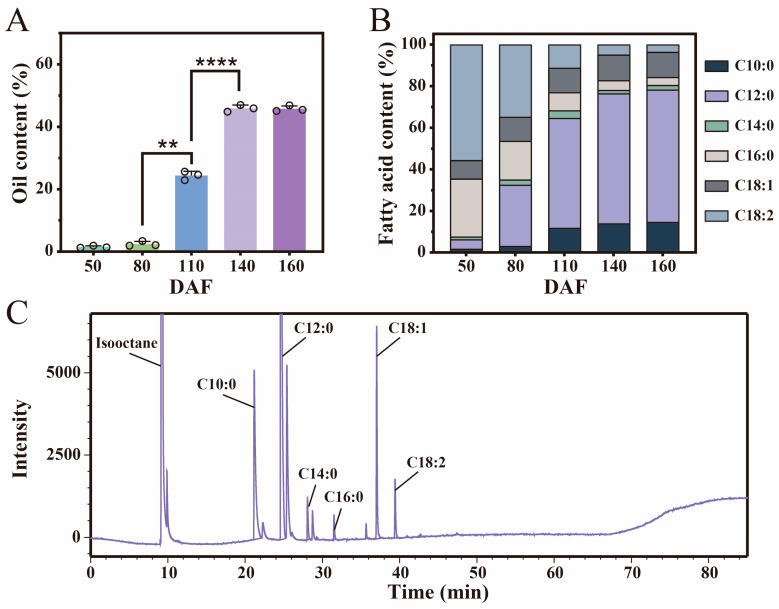
Developmental profiling of seed oil content and major fatty acids in *Litsea cubeba*. (**A**) Seed oil content at 50, 80, 110, 140, and 160 days after flowering (DAF). Values are shown as mean ± SD from three biological replicates. Asterisks indicate significant differences between developmental stages (** *p* < 0.01; **** *p* < 0.0001). (**B**) Relative proportions of the major fatty acids in *L. cubeba* seed oil, including C10:0, C12:0, C14:0, C16:0, C18:1, and C18:2. (**C**) Representative GC-FID chromatogram of fatty acid methyl esters from *L. cubeba* seed oil, with major peaks annotated.

Oil accumulation in *L. cubeba* seeds showed a clear developmental increase. At 50 and 80 DAF, oil content remained low, indicating that lipid deposition was still limited during early seed development. A marked increase was observed at 110 DAF, when oil content reached approximately one quarter of seed dry weight. Oil accumulation further increased by 140 DAF and remained at a comparable level at 160 DAF, suggesting that the main oil deposition phase occurred between 80 and 140 DAF and approached a plateau thereafter (Figure 1A).

The fatty acid profile also changed markedly during seed development. At 50 DAF, the seed oil was dominated by C18:2, whereas C12:0 accounted for only a small fraction of the total fatty acids. As development proceeded, the proportion of C12:0 increased sharply and became the dominant fatty acid from 110 DAF onward. At 140 and 160 DAF, C12:0 accounted for more than 60% of the detected major fatty acids. In contrast, C18:2 decreased continuously during seed maturation, while C10:0 and C18:1 remained as secondary components in the later stages. This shift from a C18:2-rich early profile to a C12:0-dominated mature profile indicates that lauric acid accumulation is a major feature of *L. cubeba* seed oil formation (Figure 1B).

The representative GC-FID chromatogram showed clear separation of the main fatty acid methyl ester peaks, including C10:0, C12:0, C14:0, C16:0, C18:1, and C18:2. The C12:0 peak was one of the most prominent signals in the chromatogram, consistent with the high proportion of lauric acid observed in the fatty acid composition analysis. Isooctane was detected as the solvent peak and was not included in fatty acid quantification (Figure 1C). Together, the oil accumulation pattern and the developmental shift in fatty acid composition provide a lauric acid-rich biochemical background for subsequent structure-based prioritization of FatB-like candidates.

### 3.2. Phylogenetic Placement and Conserved Motif Features of LcFatB Candidates

**Figure 2:**
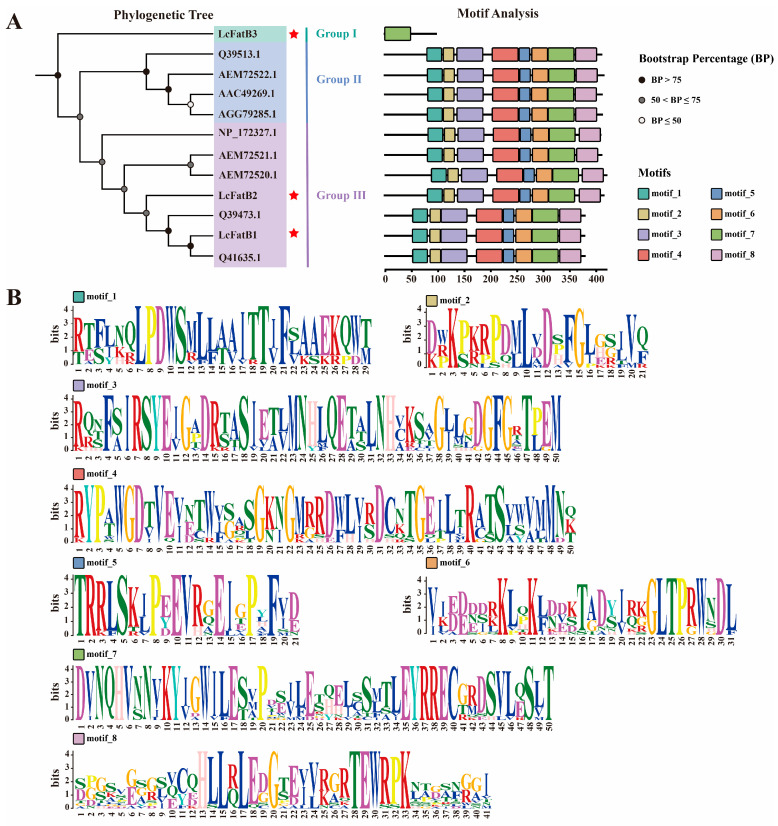
Evolutionary grouping and motif architecture of LcFatB candidates. (**A**) Phylogenetic tree and conserved motif distribution of LcFatB candidates and reference plant FatB proteins. LcFatB1, LcFatB2, and LcFatB3 are marked with red stars. Bootstrap percentages are indicated by node symbols: BP > 75, 50 < BP ≤ 75, and BP ≤ 50. Conserved motifs are shown as colored boxes and numbered motif_1 to motif_8. (**B**) Sequence logos of the eight conserved motifs identified in the FatB-related protein set. The height of each amino acid represents its relative conservation at the corresponding position.

Three FatB-like candidates were retained from the *L. cubeba* seed transcriptome assembly and designated as LcFatB1, LcFatB2, and LcFatB3. Phylogenetic analysis placed these candidates within plant FatB-related branches but showed different positions among them. LcFatB1 and LcFatB2 clustered within the same major group as several reference FatB proteins, whereas LcFatB3 was separated from the two main candidates and located in a distinct branch. This placement indicates that the three candidates are not equivalent members within the FatB-like set and that LcFatB1 and LcFatB2 are more closely related to the reference sequences used for downstream comparison (Figure 2A).

Motif analysis further distinguished LcFatB3 from LcFatB1 and LcFatB2. Most reference FatB proteins displayed a conserved motif arrangement composed of multiple repeated elements across the protein sequence. LcFatB1 and LcFatB2 retained a similar motif organization to the reference FatB proteins in the same phylogenetic group, suggesting that their FatB-like sequence architecture is relatively complete. In contrast, LcFatB3 showed a simplified motif pattern, consistent with its shorter sequence and its separated phylogenetic position (Figure 2A).

Sequence logo analysis showed that the identified motifs contained conserved residue patterns across the FatB-related sequences. Several motifs displayed high information content at specific positions, indicating that these regions are conserved within the analyzed FatB set. The conservation of these motifs supports the FatB-like identity of LcFatB1 and LcFatB2, while the reduced motif representation in LcFatB3 suggests that it is less suitable for the main MD-based comparison under the present structural workflow and should be considered as a shorter supplementary candidate (Figure 2B).

### 3.3. Structural Modeling and Sequence Conservation of LcFatB Candidates

**Figure 3:**
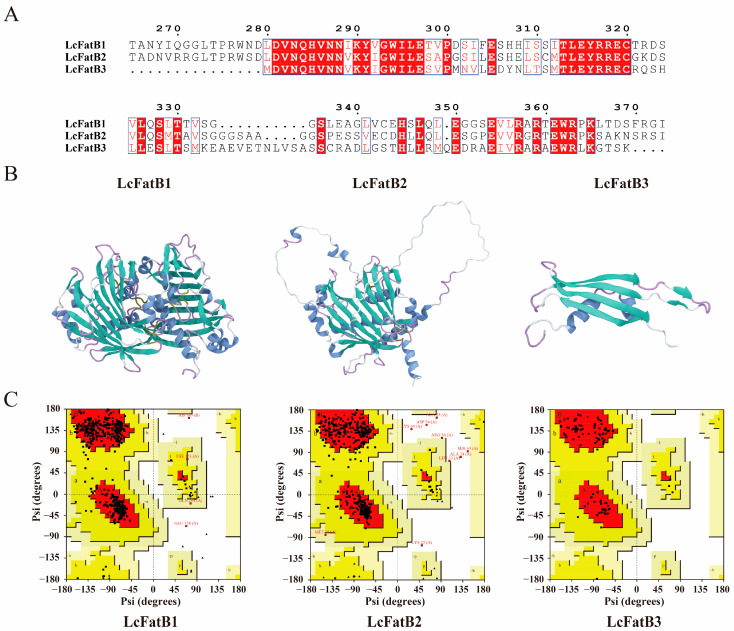
Sequence conservation, predicted structures, and model validation of LcFatB candidates. (**A**) Multiple sequence alignment of the conserved region shared by LcFatB1, LcFatB2, and LcFatB3. Conserved residues are highlighted in red, and similar residues are boxed. (**B**) Predicted three-dimensional structures of LcFatB1, LcFatB2, and LcFatB3 generated by homology modeling. (**C**) Ramachandran plot analysis of the predicted LcFatB models.

Multiple sequence alignment of the three LcFatB candidates showed that LcFatB1, LcFatB2, and LcFatB3 shared a conserved region corresponding to the C-terminal part of the modeled proteins. Within this region, several continuous residue blocks were highly conserved among the three candidates, including segments around the LDVNQHVNN, GWILE, and TLEYRREC motifs. LcFatB1 and LcFatB2 showed stronger overall similarity across this region, whereas LcFatB3 contained terminal gaps and a shorter aligned segment, consistent with its reduced sequence length (Figure 3A).

Homology modeling further separated the three candidates at the structural level. LcFatB1 formed a larger FatB-like fold with an expanded β-sheet-rich core and surrounding helices, whereas LcFatB2 retained a comparable core region but contained more extended loop segments. In contrast, the LcFatB3 model was markedly shorter and lacked several structural elements present in LcFatB1 and LcFatB2. This difference in model completeness indicates that LcFatB1 and LcFatB2 provide more suitable structural models for subsequent comparative docking and dynamic analyses under the present workflow, whereas LcFatB3 was retained as a shorter supplementary candidate with lower confidence for downstream dynamic comparison (Figure 3B).

Ramachandran plot analysis was used to check the stereochemical reasonability of the three predicted models. The residue distributions were mainly concentrated in favored and allowed regions, although limited outliers were present in the modeled structures (Figure 3C).

Pairwise alignment between LcFatB1 and LcFatB2 showed broad sequence similarity across most aligned regions. Conserved residue blocks were distributed throughout the alignment, whereas sequence gaps and divergent residues were mainly observed in discrete regions rather than across the entire sequence. This pattern supports the use of LcFatB1 and LcFatB2 as the main pair for subsequent comparison of C12 substrate accommodation, residue–ligand contacts, and tunnel geometry (Figure S1). The predicted ligand-contacting residues identified later in the docking and MD analyses were therefore interpreted as candidate sites derived from the present structural workflow, rather than as experimentally validated determinants of C12 specificity.

### 3.4. Docking of LcFatB Candidates with the C12 Acyl-4′-Phosphopantetheine Surrogate

**Figure 4:**
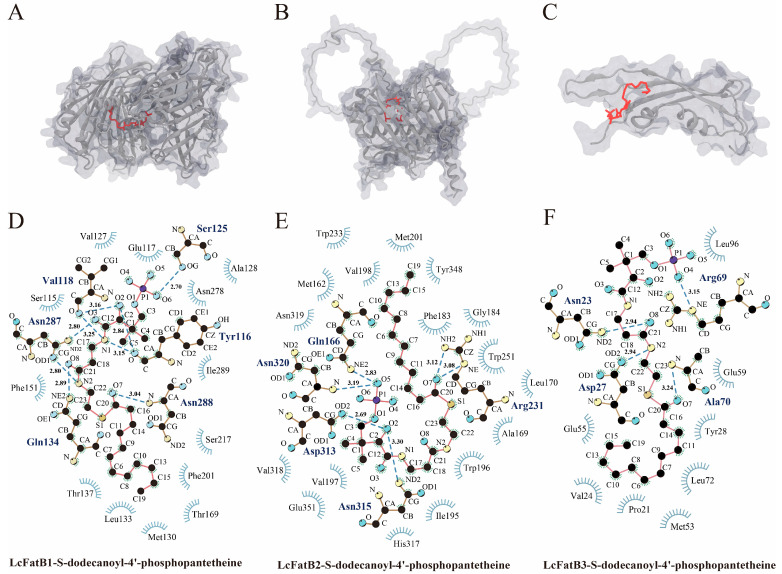
Predicted binding modes and interaction patterns of LcFatB candidates with the C12 substrate surrogate. (**A**–**C**) Docking poses of S-dodecanoyl-4′-phosphopantetheine in the predicted pocket regions of LcFatB1, LcFatB2, and LcFatB3. Protein structures are shown in gray, and the ligand is shown in red. (**D**–**F**) Two-dimensional interaction diagrams of S-dodecanoyl-4′-phosphopantetheine with LcFatB1, LcFatB2, and LcFatB3, respectively.

Docking analysis showed that S-dodecanoyl-4′-phosphopantetheine could be accommodated in the predicted pocket region of all three LcFatB models. In LcFatB1, the ligand was positioned within a relatively enclosed cavity, with the phosphopantetheine region surrounded by several polar residues and the dodecanoyl chain extending into the interior of the pocket. In LcFatB2, the ligand also occupied the central pocket region, although the surrounding loop architecture appeared more open. In LcFatB3, the ligand was located along a shallower binding groove, consistent with the shorter modeled structure of this candidate (Figure 4A–C).

Two-dimensional interaction maps further indicated different residue–ligand contact patterns among the three candidates. In LcFatB1, S-dodecanoyl-4′-phosphopantetheine formed multiple polar contacts with residues around the phosphopantetheine and thioester regions, including Ser125, Tyr116, Asn278, Asn287, Asn288, and Gln134. Several surrounding residues, such as Val127, Ala128, Phe151, Leu133, Thr169, Met130, Ile289, and Phe201, were positioned near the ligand and may contribute to pocket packing around the acyl chain (Figure 4D). This contact pattern is consistent with a more constrained predicted pocket environment for C12 substrate placement in LcFatB1.

LcFatB2 retained a distinct interaction pattern. The ligand was contacted by residues including Gln166, Asn320, Asp313, Asn315, and Arg231, while Val198, Met201, Phe183, Gly184, Trp196, Trp251, Tyr348, and Gln351 were distributed around the acyl chain or phosphopantetheine arm (Figure 4E). These contacts indicate that LcFatB2 can also accommodate the C12 surrogate, but the binding environment differs from that of LcFatB1. In LcFatB3, fewer pocket-forming residues were observed around the ligand, with polar contacts mainly involving Asn23, Asp27, Arg69, and Ala70 (Figure 4F). Together, the docking results provided an initial structure-based basis for selecting LcFatB1 and LcFatB2 as the main candidates for subsequent dynamic comparison, while LcFatB3 was retained as a shorter supplementary candidate with limited structural interpretability in this workflow. These docking results were interpreted as predicted pocket-compatibility evidence rather than direct evidence of FatB catalytic activity.

### 3.5. Dynamic Stability of LcFatB1 and LcFatB2 C12-Bound Complexes

**Figure 5:**
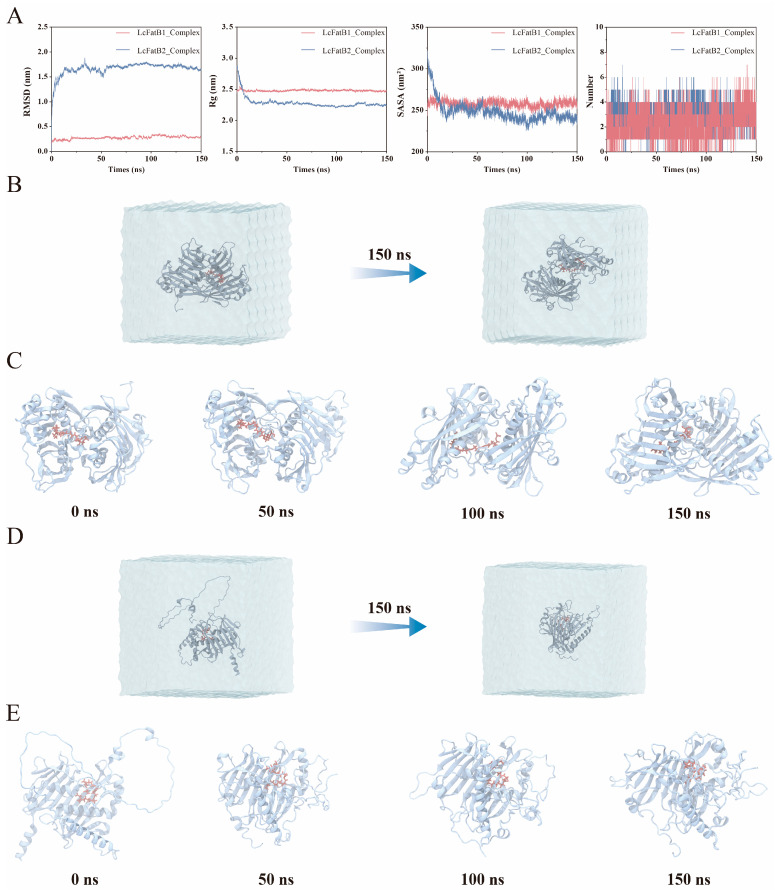
Molecular dynamics behavior and conformational snapshots of LcFatB1 and LcFatB2 complexes. (**A**) Time-dependent profiles of backbone RMSD, radius of gyration (Rg), solvent-accessible surface area (SASA), and protein–ligand hydrogen bond number during 150 ns simulations. (**B**) Solvated simulation-box snapshots of the LcFatB1 complex at the initial and final simulation states. (**C**) Representative conformations of the LcFatB1 complex extracted at 0, 50, 100, and 150 ns. (**D**) Solvated simulation-box snapshots of the LcFatB2 complex at the initial and final simulation states. (**E**) Representative conformations of the LcFatB2 complex extracted at 0, 50, 100, and 150 ns. The protein is shown in light blue, and S-dodecanoyl-4′-phosphopantetheine is shown in red.

The dynamic behavior of LcFatB1 and LcFatB2 bound to S-dodecanoyl-4′-phosphopantetheine was evaluated by 150 ns molecular dynamics simulations. The LcFatB1 complex showed low backbone RMSD values during most of the trajectory. After the initial equilibration period, the RMSD of LcFatB1 mainly fluctuated around 0.28 nm, with most values distributed within 0.24–0.33 nm from 10 to 150 ns. In contrast, the LcFatB2 complex showed a larger conformational displacement and then reached a stable high-RMSD state, with an average RMSD of approximately 1.69 nm and most values distributed within 1.59–1.77 nm during the same period. This pattern suggests that the higher RMSD of LcFatB2 mainly reflected early conformational relaxation from the starting model rather than continuous structural drift (Figure 5A).

The compactness-related descriptors further distinguished the two systems. LcFatB1 maintained a stable Rg profile around 2.48 nm from 10 to 150 ns, indicating that the overall compactness of the complex remained stable during the simulation. LcFatB2 stabilized at a lower Rg level, with an average value of approximately 2.26 nm and most values within 2.22–2.31 nm during the same period. The SASA of LcFatB1 mainly fluctuated around 258 nm^2^, with most values distributed within 252–264 nm^2^, whereas LcFatB2 showed a lower average SASA of approximately 246 nm^2^, with most values within 235–257 nm^2^. These compactness-related changes are consistent with rearrangement of the extended loop regions in the LcFatB2 model, in which initially loose peripheral segments may pack closer to the protein core. The protein–ligand hydrogen bond number averaged approximately 2.3 in the LcFatB1 complex and 2.7 in the LcFatB2 complex from 10 to 150 ns, with most values ranging from 1 to 4 in both systems. These results indicate that ligand stabilization in both complexes was maintained through dynamic residue contacts rather than a fixed hydrogen-bonding pattern (Figure 5A).

Structural snapshots provided a direct view of conformational evolution during the simulations. In the LcFatB1 complex, the overall fold was retained from 0 to 150 ns, and the ligand remained located near the internal pocket region across the selected time points (Figure 5B,C). In the LcFatB2 complex, the protein also retained the ligand in the modeled binding region, but the surrounding flexible segments showed more visible positional changes during the trajectory (Figure 5D,E). Together, these results indicate that both complexes reached stable C12-bound states, but LcFatB1 retained a lower-displacement conformation, whereas LcFatB2 stabilized the ligand after a larger initial structural relaxation. To further examine local flexibility, residue-level Cα RMSF profiles were calculated for both complexes and provided in Figure S2. In LcFatB1, most modeled core regions showed relatively low fluctuations, whereas higher RMSF values were mainly observed in terminal or peripheral loop regions. LcFatB2 displayed larger fluctuations in several flexible segments, consistent with its larger conformational relaxation observed in the RMSD profile and structural snapshots. Additional backbone-aligned superimposed views of the 0 ns and 150 ns conformations are provided in Figure S3, further illustrating the initial-to-final conformational changes in the two C12-bound complexes.

### 3.6. Free Energy Landscape Reveals Distinct Conformational States

**Figure 6:**
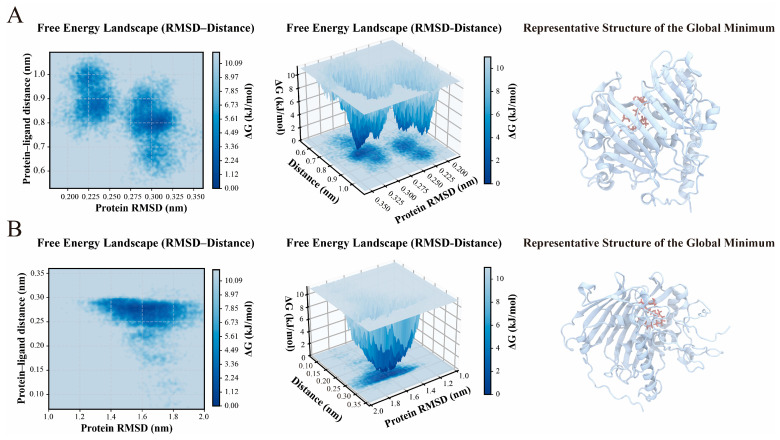
Free energy landscapes and global-minimum structures of the C12-bound LcFatB complexes. (**A**) Two-dimensional and three-dimensional free energy landscapes of the LcFatB1 complex constructed using protein RMSD and protein–ligand distance as reaction coordinates, together with the representative structure extracted from the global minimum basin. (**B**) Two-dimensional and three-dimensional free energy landscapes of the LcFatB2 complex constructed using the same reaction coordinates, together with the representative structure extracted from the global minimum basin. The protein is shown in light blue, and S-dodecanoyl-4′-phosphopantetheine is shown in red.

Free energy landscapes were constructed using protein RMSD and protein–ligand distance as reaction coordinates to examine the dominant conformational states sampled by the two C12-bound complexes. The LcFatB1 complex showed low-energy regions within a low protein-RMSD range, consistent with its limited global displacement during the MD trajectory. Its dominant low-energy basin was associated with protein–ligand distances of approximately 0.8–1.0 nm, indicating that the C12 surrogate remained retained in the internal pocket while the protein maintained a conformation close to the starting model (Figure 6A).

The LcFatB2 complex displayed a distinct free-energy distribution. Its low-energy basin occurred at a higher protein-RMSD range, consistent with the larger conformational relaxation observed in the RMSD profile. However, the corresponding protein–ligand distance was shorter and more concentrated, mainly around 0.25–0.30 nm (Figure 6B). This result indicates that LcFatB2 did not remain unstable after the initial relaxation; instead, it converged into a different C12-bound conformational state. Therefore, the shorter protein–ligand distance in LcFatB2 should be interpreted as local ligand retention within a rearranged pocket environment rather than direct evidence of stronger C12 specificity.

The representative structures extracted from the global minimum basins further supported this distinction. LcFatB1 retained a compact FatB-like fold with the C12 surrogate positioned in the internal binding region, whereas LcFatB2 maintained the ligand near the pocket after rearrangement of surrounding peripheral segments. These results show that both candidates can stabilize the C12 surrogate, but through different conformational states. The extracted global-minimum structures were therefore used for subsequent residue–ligand interaction and tunnel analyses.

### 3.7. Top-Ranked Residue Contacts Associated with C12 Surrogate Stabilization

Residue–ligand interaction analysis was performed using the global-minimum representative structures and the corresponding MD trajectories. To compare the local ligand-stabilizing networks between the two complexes, two complementary descriptors were used: hydrogen bond occupancy, which reflects the persistence of polar contacts, and minimum heavy-atom distance, which reflects the spatial proximity of selected residues to S-dodecanoyl-4′-phosphopantetheine during the trajectory.

**Figure 7:**
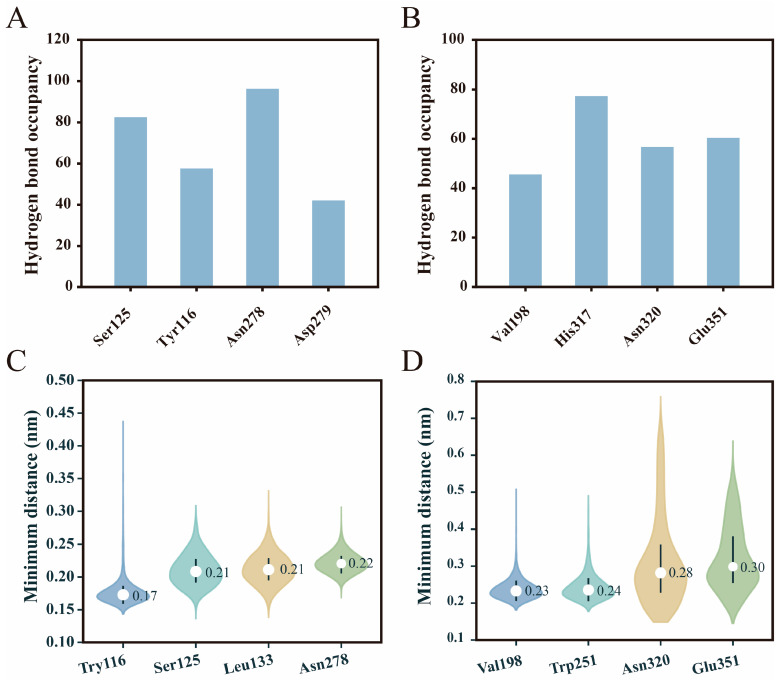
Top-ranked residue interactions in the global-minimum LcFatB complexes. (**A**) Four residues with the highest hydrogen bond occupancy in the LcFatB1 complex. (**B**) Four residues with the highest hydrogen bond occupancy in the LcFatB2 complex. (**C**) Four residues with the shortest average minimum heavy-atom distances to S-dodecanoyl-4′-phosphopantetheine in the LcFatB1 complex during the MD trajectory. (**D**) Four residues with the shortest average minimum heavy-atom distances to S-dodecanoyl-4′-phosphopantetheine in the LcFatB2 complex during the MD trajectory.

In the LcFatB1 complex, the four residues with the highest hydrogen bond occupancy were Asn278, Ser125, Tyr116, and Asp279. These residues were mainly located around the phosphopantetheine and thioester regions of the C12 surrogate, suggesting that polar contacts in LcFatB1 were concentrated near the chemically reactive portion of the ligand (Figure 7A). Minimum-distance analysis further supported this local compactness. Tyr116 showed the shortest average distance to the ligand, and Ser125, Leu133, and Asn278 also remained within a narrow distance range of approximately 0.21–0.22 nm (Figure 7C). The overlap between the hydrogen-bonding and proximity-ranked residues, especially Tyr116, Ser125, and Asn278, indicates a compact and persistent contact environment in the LcFatB1 pocket.

LcFatB2 showed a different interaction profile. His317, Glu351, Asn320, and Val198 ranked as the top hydrogen-bonding residues, whereas Val198, Trp251, Asn320, and Glu351 showed the shortest average minimum distances to the ligand (Figure 7B,D). This result indicates that LcFatB2 also retained ligand-associated contacts during the trajectory, but the polar-contact and proximity-ranked residues were distributed in a different local environment from that of LcFatB1. In particular, Val198 and Trp251 contributed mainly through close spatial proximity, whereas His317, Glu351, and Asn320 contributed to polar-contact stabilization.

Together, the residue-contact analysis shows that both LcFatB1 and LcFatB2 can maintain contacts with the C12 surrogate, but the two candidates use different local stabilizing networks. LcFatB1 is characterized by a more compact contact set involving Tyr116, Ser125, and Asn278 near the ligand, whereas LcFatB2 stabilizes the ligand through a more rearranged contact pattern involving His317, Glu351, Asn320, Val198, and Trp251. This distinction supports the interpretation that LcFatB1 and LcFatB2 do not simply differ in binding stability, but in the local organization of C12 surrogate accommodation.

### 3.8. Main Tunnel Geometry in Global-Minimum Structures Distinguishes LcFatB1 from LcFatB2

**Figure 8:**
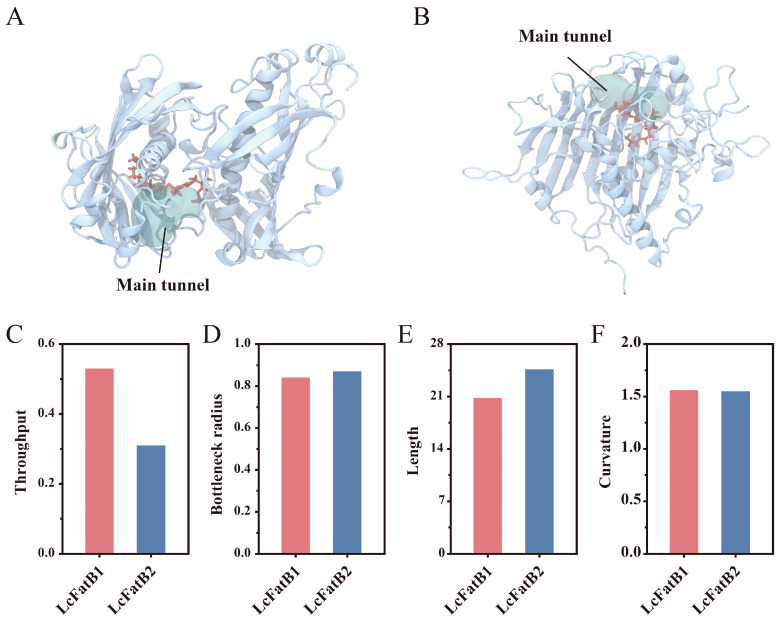
Main tunnel geometry of the global-minimum LcFatB1 and LcFatB2 complexes. (**A**) Main tunnel identified in the global-minimum representative structure of the LcFatB1 complex. (**B**) Main tunnel identified in the global-minimum representative structure of the LcFatB2 complex. The protein is shown in light blue, S-dodecanoyl-4′-phosphopantetheine is shown in red, and the main tunnel is shown as a translucent surface. (**C**–**F**) Comparison of main tunnel throughput, bottleneck radius, length, and curvature between LcFatB1 and LcFatB2.

Catalytic tunnel analysis was performed using the global-minimum representative structures of the LcFatB1 and LcFatB2 complexes. In both proteins, a main tunnel connected the ligand-binding region with the protein surface, suggesting a possible route for C12 substrate access or product release in the representative C12-bound conformations. The main tunnel of LcFatB1 was located close to the bound S-dodecanoyl-4′-phosphopantetheine and passed through a compact region near the binding pocket, whereas the corresponding tunnel in LcFatB2 extended through a different peripheral region of the folded structure (Figure 8A,B).

Quantitative tunnel descriptors further separated the two global-minimum conformations. LcFatB1 showed a higher throughput value than LcFatB2, indicating a more favorable overall tunnel score in the representative conformation (Figure 8C). The bottleneck radius was similar between the two tunnels, with LcFatB2 showing a slightly larger value (Figure 8D). By contrast, the main tunnel of LcFatB1 was shorter than that of LcFatB2, whereas their curvature values were nearly comparable (Figure 8E,F). These results indicate that the main difference between the two tunnels was not the narrowest radius or bending degree, but the combined accessibility reflected by throughput and the shorter access path in LcFatB1.

When interpreted together with the docking, MD, free energy, and residue–ligand interaction results, the tunnel descriptors are consistent with a more favorable predicted access geometry for LcFatB1 in the representative global-minimum state. LcFatB2 retained a detectable main tunnel, but its lower throughput and longer route indicate a less favorable access geometry under the same conformational-state-specific analysis. Because these tunnel descriptors were calculated from representative global-minimum structures, they should be interpreted as local conformational comparisons rather than trajectory-wide tunnel properties. Therefore, the tunnel results provide supporting structural evidence for candidate prioritization but do not by themselves establish a persistent dynamic access route or functional superiority of LcFatB1.

## 4. Discussion

The seed oil profile of *L. cubeba* provides the biochemical context for FatB candidate prioritization. During seed development, oil content increased markedly after the early stage, while the fatty acid profile shifted from a C18:2-rich pattern to a C12:0-dominated composition. Developmental shifts in seed oil accumulation and fatty acid profiles have also been reported in other oilseed systems, where maturation stage and lipid metabolic remodeling strongly influence the final fatty acid composition [27,28]. This developmental transition is consistent with previous reports that *L. cubeba* seed oil is characterized by a high proportion of lauric acid and can serve as a plant source of medium-chain fatty acids. In plastidial fatty acid synthesis, acyl-ACP thioesterases terminate acyl-chain elongation by releasing fatty acids from acyl-ACP substrates. Because FatB-type thioesterases are generally associated with saturated acyl-ACP hydrolysis, they provide a biologically reasonable entry point for prioritizing candidates potentially related to C12:0 accumulation in *L. cubeba*. However, the high C12:0 content observed at the seed level cannot by itself identify the enzyme or enzymes contributing to this phenotype. Candidate prioritization therefore requires sequence, structural, and substrate-accommodation evidence.

The three LcFatB candidates showed FatB-like features, but their sequence organization and structural completeness differed. LcFatB1 and LcFatB2 were closer to representative plant FatB proteins in the phylogenetic tree, retained more complete motif architectures, and formed more interpretable FatB-like structural models. LcFatB3 was separated from these two candidates, contained fewer conserved elements, and produced a markedly shorter model. These observations indicate that the three transcriptome-derived candidates should not be treated as equivalent structural targets. LcFatB1 and LcFatB2 are more suitable for comparative structural analysis, whereas LcFatB3 is better interpreted as a shorter supplementary candidate under the present workflow. This interpretation does not exclude a biological role for LcFatB3, but its incomplete structural representation limits the confidence of downstream docking and dynamic comparison.

Sequence annotation alone is insufficient to infer whether a FatB-like candidate is associated with C12 acyl-substrate accommodation or C12:0 accumulation. Productive accommodation of a C12 acyl substrate depends on pocket shape, residue placement, the positioning of the thioester region, and possible substrate-access routes. In this context, the use of S-dodecanoyl-4′-phosphopantetheine was intended to retain key chemical features of the acyl-ACP substrate, including the dodecanoyl chain, thioester linkage, and phosphopantetheine arm, while avoiding the complexity of full acyl-ACP docking. This surrogate therefore provides a more relevant ligand model than free lauric acid for comparing pocket compatibility, although it still cannot fully reproduce protein–protein recognition between ACP and FatB. Docking showed that all three candidates could accommodate the C12 surrogate, but LcFatB1 and LcFatB2 provided more interpretable pocket-bound poses than LcFatB3. In particular, LcFatB1 placed the ligand in a more enclosed pocket, whereas LcFatB2 accommodated the ligand in a more open local environment.

MD simulations further separated the two main candidates, but these results should not be interpreted as indicating that LcFatB2 is a poor or nonfunctional FatB-like protein. LcFatB1 maintained a lower RMSD range and a compact C12-bound state, whereas LcFatB2 showed a larger conformational relaxation before reaching a stable high-RMSD state. The decrease in Rg and SASA of LcFatB2 after the initial relaxation suggests that this complex underwent structural compaction rather than continuous destabilization. The free energy landscape also supported this interpretation. LcFatB2 sampled its low-energy basin at a higher protein RMSD but with a shorter and more concentrated protein–ligand distance, whereas LcFatB1 remained closer to the starting conformation. Therefore, the shorter protein–ligand distance in LcFatB2 should be interpreted as local ligand retention within a rearranged pocket environment, rather than direct evidence of stronger C12 specificity. Productive C12 accommodation depends not only on ligand proximity, but also on the orientation of the thioester region, pocket packing around the acyl chain, persistence of residue contacts, and substrate-access geometry. In MD-based protein–ligand analysis, trajectory-derived interaction descriptors are useful for interpreting ligand retention, contact persistence, and binding-state evolution beyond static docking poses [29]. Free-energy landscape analysis can further help identify dominant conformational basins and interpret protein–ligand binding states sampled during MD simulations [30].

Residue-contact analysis further indicated that LcFatB1 and LcFatB2 used different local stabilizing networks. In LcFatB1, Asn278, Ser125, and Tyr116 ranked highly in hydrogen-bond occupancy or proximity to the ligand, indicating a compact contact environment around the phosphopantetheine and thioester regions. In LcFatB2, His317, Glu351, Asn320, Val198, and Trp251 contributed to ligand association, but these contacts were distributed in a different pocket environment and showed greater distance heterogeneity. These results indicate that both LcFatB1 and LcFatB2 can accommodate the C12 surrogate, but they do so through different structural solutions. LcFatB1 is favored in the present prioritization because its docking pose, lower global displacement, compact local contact set, and access-route geometry jointly support a more constrained C12-binding environment. LcFatB2 remains a plausible FatB-like candidate rather than an excluded candidate. It may represent a structurally distinct thioesterase with a different chain-length preference, such as toward C14 or C16 acyl substrates, but this possibility was not tested in the present study. Recent studies of acyl-ACP thioesterase-based fatty acid production also indicate that chain-length selectivity requires substrate- or product-level experimental evaluation, rather than inference from a single candidate sequence or structure alone [31].

Tunnel analysis added another level of comparison between LcFatB1 and LcFatB2. Although both proteins contained a main tunnel connecting the binding region with the protein surface, LcFatB1 showed higher throughput and a shorter tunnel length, while bottleneck radius and curvature were similar between the two proteins. These features suggest a more favorable access route in the global-minimum representative conformation of LcFatB1. However, this interpretation should be restricted to the representative conformational state analyzed here, because tunnel geometry can vary during protein breathing, side-chain rearrangement, and loop movement. The present tunnel analysis therefore provides a conformational-state-specific comparison rather than a trajectory-wide description of tunnel dynamics. Future trajectory-wide or cluster-based tunnel analysis would be needed to determine whether this access-route difference is maintained across the dominant conformational ensemble. Such trajectory-wide tunnel analyses are relevant because ligand transport pathways can vary with protein conformational dynamics and are increasingly used in enzyme interpretation and rational design [32].

Taken together, the developmental fatty acid profile, sequence features, docking behavior, MD trajectory, free energy landscape, residue–ligand contacts, and tunnel descriptors support LcFatB1 as the strongest structurally prioritized FatB candidate among the three transcriptome-derived proteins examined here. This conclusion should be interpreted as candidate prioritization rather than direct functional confirmation. The present analysis does not exclude the possibility that LcFatB2, other FatB-like members, or candidates not recovered from the current transcriptome assembly may also contribute to C12:0 synthesis in vivo. Recent plant candidate-gene studies also emphasize that sequence-based identification or expression-based inference should be followed by functional assays, genetic manipulation, or targeted validation before assigning biological function [33,34,35,36,37]. Biochemical assays using acyl-ACP or suitable substrate surrogates, heterologous expression, subcellular localization, substrate-chain-length comparison, and site-directed mutagenesis are still required to define the catalytic role and substrate preference of LcFatB1. The predicted LcFatB1 contact residues, including Tyr116, Ser125, and Asn278, should also be interpreted with caution. Previous studies of plant acyl-ACP thioesterases have shown that substrate specificity is affected by residues shaping the acyl-binding pocket, pocket depth, and the spatial organization of the two hotdog domains. The C12-associated *U. californica* FatB has been structurally characterized as a lauroyl-ACP thioesterase, and its structure provides a reference framework for examining C12 acyl-chain accommodation in FatB-type enzymes. Structural and mutational analyses of FatB enzymes from *U. californica* and *Cuphea* species further support the view that local pocket residues and domain-level arrangements can alter acyl-chain selectivity. In the present study, Tyr116, Ser125, and Asn278 were identified from docking and MD-based contact analyses of LcFatB1. These residues therefore provide testable candidate sites for future mutagenesis, but they should not yet be regarded as experimentally validated determinants of lauroyl-ACP specificity.

## 5. Conclusions

This study combined developmental fatty acid profiling with structure-based analysis to prioritize FatB-like thioesterase candidates in *L. cubeba*. The increase in C12:0 during seed development provided the biochemical background for examining FatB candidates potentially involved in medium-chain acyl-substrate release. Among the three candidates retrieved from the seed transcriptome assembly, LcFatB1 and LcFatB2 showed more complete FatB-like sequence and structural features than LcFatB3. Docking with S-dodecanoyl-4′-phosphopantetheine, followed by MD simulation, free energy landscape analysis, residue–ligand contact profiling, and tunnel analysis, further distinguished the two main candidates. Under the present structure-based workflow, LcFatB1 showed a more enclosed C12-binding environment, lower conformational displacement, persistent contacts involving Tyr116, Ser125, and Asn278, and a main tunnel with higher throughput and shorter length in the global-minimum representative conformation. These features support LcFatB1 as the structurally prioritized candidate for future biochemical testing, while LcFatB2 remains a plausible FatB-like candidate with a distinct C12-bound state. This prioritization does not exclude the possible contribution of LcFatB2, other FatB-like members, or candidates not recovered from the current transcriptome assembly to C12:0 accumulation in vivo. The findings provide candidate-level structural clues that may guide subsequent functional assays and future efforts to understand or engineer lauric acid-rich oil traits in *L. cubeba*. Recent metabolic engineering studies also highlight the value of acyl-carrier-linked routes for producing medium-chain oleochemicals [38]. However, this prioritization is based on computational evidence and should not be interpreted as direct functional confirmation. Enzymatic assays using acyl-ACP or suitable substrate surrogates, heterologous expression, substrate-chain-length comparison, and site-directed mutagenesis are still required to define the catalytic activity and substrate preference of LcFatB1.

## Data Availability

The data supporting the findings of this study are available within the article and its Supplementary Materials. The raw transcriptome reads of *Litsea cubeba* seeds are available from the Genome Sequence Archive of the China National Center for Bioinformation under accession number CRA021915. The FatB candidate sequences analyzed in this study were retrieved from the de novo seed transcriptome assembly generated from these raw reads.

## Supplementary Materials

The following supporting information can be downloaded at: https://www.mdpi.com/article/10.3390/biom16060813/s1, Figure S1: Pairwise sequence alignment of LcFatB1 and LcFatB2. Figure S2: Residue-level RMSF profiles of LcFatB1 and LcFatB2 complexes during 150 ns molecular dynamics simulations. Figure S3: Superimposed views of the initial and final conformations of LcFatB complexes during 150 ns molecular dynamics simulations.
