# Supplementary material for: Structural Prioritization of FatB Thioesterase Candidates Potentially Related to Lauric Acid-Rich Seed Oil in Litsea cubeba
Source: Biomolecules. 2026 May 30;16(6):813. doi: 10.3390/biom16060813 (PMC13297048; doi:10.3390/biom16060813)
Supplement: Supplementary file 1 [file biomolecules-16-00813-s001.zip › biomolecules-4316850-supplementary.pdf]

Supporting Information

Structural Prioritization of FatB Thioesterase Candidates Potentially Related to Lauric Acid-Rich Seed Oil in *Litsea cubeba*

Wenyan Yuan [1,2], Changzhu Li [1,2,3,4], Jingzhen Chen [2,4], Peiwang Li [2], Xiao Zhou [2,4], Wei Wu [1,2], Lijuan Jiang [1], Wenbin Zeng [1], Feng Wen [1], Yunzhu Chen [2,4,*] and Yan Yang [2,4,*]

[1] State Key Laboratory of Woody Oil Resources Utilization, Central South University of Forestry and Technology, 498 South Shaoshan Road, Changsha 410004, China; 20231100133@csuft.edu.cn (W.Y.); slky@hnlky.cn (C.L.); w2052065326@163.com (W.W.); znljiang2542@163.com (L.J.); csuft85658537@163.com (W.Z.); wen19992022@163.com (F.W.)

[2] Hunan Academy of Forestry, Changsha 410004, China; chenjingzhen602@hnlky.cn (J.C.); lindan523@163.com (P.L.); zx924647234@163.com (X.Z.)

[3] Institute of Urban Agriculture, Chinese Academy of Agricultural Sciences, Chengdu 610213, China

[4] Yuelushan Laboratory, Changsha 410000, China

* Correspondence: cyzcarol@foxmail.com (Y.C.); yangyanzupei@126.com (Y.Y.)

> LcFatB1

MATTSLASAFCSMKAVMLARDGRGMKPRSSDLQLRAGNAQTSLKMINGTKF
SDTESLKRLPDWSMLFTVITTIFSAAEKQWTNLEWKPKPKPPHLLDDHFGLH
GLVFRRTFAIRSYEVGPDRSISIVAVMNHLQEATLNHAESVGILGDGFGETLEM
SKRDLMWVVRRTHVAVERYPAWGDTVEVESWIGASGNNGMRREFLVRDCKT
GEILTRCTSLSVLMNTRTRRLSKIPEEVRGEIGPVFIDKVAVKDEEIKKLQKLNH
STANYIQGGLTPRWNDLDVNQHVNNIKYVGWILETVPDSIFESHHISSITLEYR
RECTRDSVLQSLTTVSGGSLEAGLVCEHSLQLEGGSEVLRARTEWRPKLTDSF
RGISVIPAEPSV

> LcFatB2

MVATAAASAFFPVGAPATSSATSAKASMMPDNLDARGIKPKPASSSGLQVKAN
AHASPKINGSKVSTDTLKGDDTLTSSPAPRTFINQLPDWSMFLAAITTIFLAAE
KQWTNLDWKPRRPDMLADPFGIGRFMQDGLIFRQHFAIRSYEIGADRTASIET
LMNHLQETALNHVRSAGLLGDGFGATPEMSRRDLIWVVTRMQVLVDRYPAW
GDIVEVETWVGASGKNGMRRDWLVRDSQTGEILTRATSVWVMMNKRTRRLS
KIPEEVRGEIGPYFMENVAIIEEDSRKLQKLNENTADNVRRGLTPRWSDLDVN
QHVNNVKYIGWILESAPGSILESHELSCMTLEYRRECGKDSVLQSMTAVSGGG
SAAGGSPESSVECDHLLQLESGPEVVRGRTEWRPKSAKNSRSILEMPAESS

> LcFatB3

MDVNQHVNNVKYIGWILESVPMNVLEDYNLTSMTLEYRRECRQSHLLESLTS
MKEAEVETNLVSASSCRADLGSTHLLRMQEDRAEIVRARAEWRLKGTSK

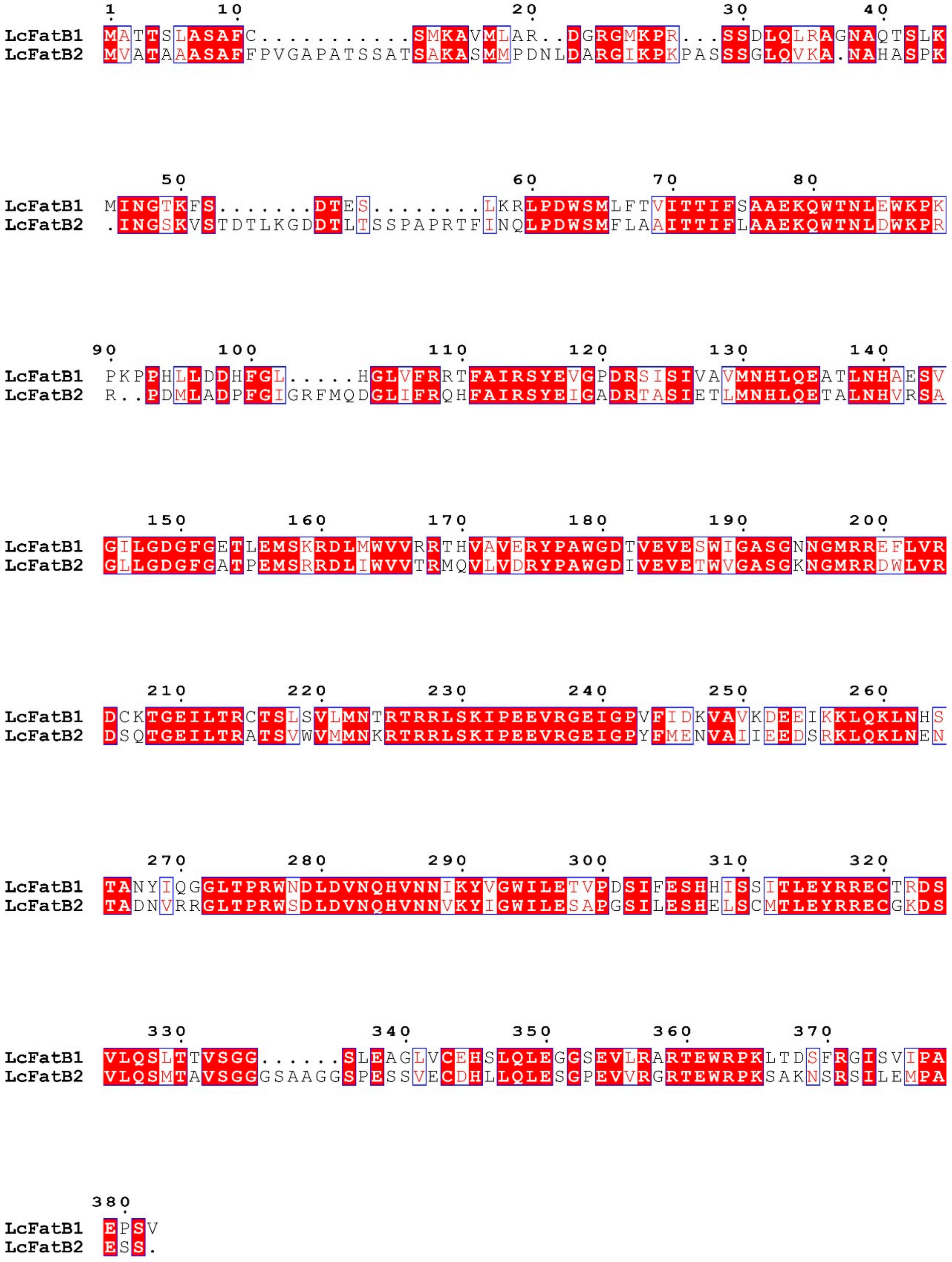

Figure S1. Pairwise sequence alignment of LcFatB1 and LcFatB2. Conserved residues are highlighted in red, and similar residues are boxed.

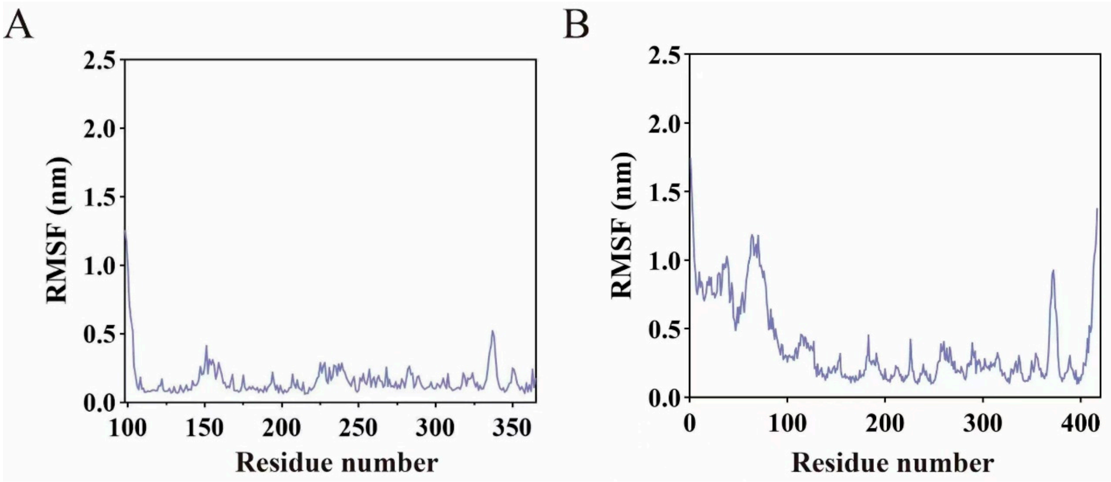

Figure S2. Residue-level RMSF profiles of LcFatB1 and LcFatB2 complexes during 150 ns molecular dynamics simulations. (A) Cα RMSF profile of the LcFatB1 – S-dodecanoyl-4′ -phosphopantetheine complex. (B) Cα RMSF profile of the LcFatB2 – S-dodecanoyl-4′ -phosphopantetheine complex. RMSF values were calculated by residue number to evaluate local flexibility along the protein sequence. Regions with elevated RMSF mainly corresponded to terminal or flexible loop segments, whereas most modeled core regions showed lower fluctuations.

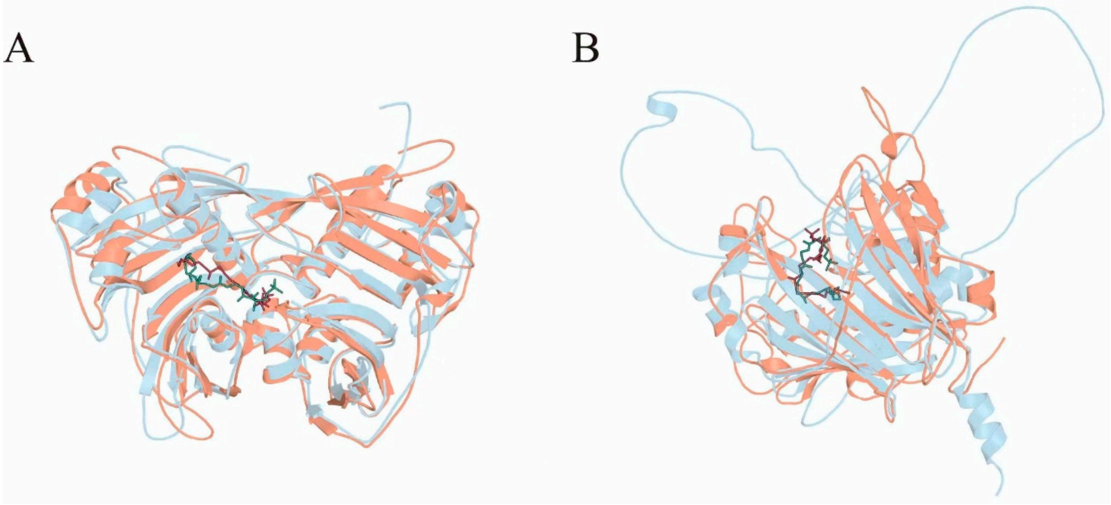

Figure S3. Superimposed views of the initial and final conformations of LcFatB complexes during 150 ns molecular dynamics simulations. (A) Backbone-aligned superimposed structure of the LcFatB1–S-dodecanoyl-4′-phosphopantetheine complex at 0 ns and 150 ns. (B) Backbone-aligned superimposed structure of the LcFatB2–S-dodecanoyl-4′-phosphopantetheine complex at 0 ns and 150 ns. The protein structures at 0 ns and 150 ns are shown in blue and red, respectively. The ligand conformations at 0 ns and 150 ns are shown in green and red, respectively.
